# Diverging from the traditional RCT paradigm

**DOI:** 10.1007/s12471-024-01858-8

**Published:** 2024-02-22

**Authors:** Pim van der Harst

**Affiliations:** Department of Cardiology, Division of Heart & Lungs, University Medical Center Utrecht, Utrecht, The Netherlands

In this issue of the *Netherlands Heart Journal*, the potential of Routine Health Care Data (RHCD) and alternative research methodologies in advancing cardiovascular care is compellingly illustrated.

Handoko emphasises the high value but considerable challenges of randomised controlled trials (RCTs) in clinical research [1]. He acknowledges RHCD as a cost-effective and efficient alternative for large-scale studies and pharmacovigilance, despite issues related to data linkage and privacy.

Bosch’s work exemplifies a practical application of RHCD to study the value of emergency CT for head trauma in patients presenting with out-of-hospital cardiac arrest due to STEMI [2]. This study observed that performing CT of the head, though not significantly altering the diagnosis or treatment plan for intracranial haemorrhage, does delay PCI treatment in STEMI patients. This observation could reshape protocols in the emergency department.

Hilt and colleagues’ exploration of socioeconomic status (SES) disparities in myocardial infarction care, utilising claims data, offers a different yet equally important angle not provided by RCTs [3].

Gawałko’s study shifts focus to the implementation of innovative healthcare delivery models [4]. The TeleCheck-AF approach demonstrates a significant shift in real-life healthcare utilisation while maintaining high levels of patient satisfaction and prompts a re-evaluation of reimbursement codes.

These studies, all diverging from the traditional RCT paradigm, collectively contribute to a broader understanding of cardiovascular care. They highlight how alternative data collection and research methodologies can provide unique and important insights into patient care, treatment efficacy, and healthcare system efficiencies. These examples also show that the diversification of research approaches in cardiovascular medicine is not only feasible but also essential for advancing the care for our patients.
